# A *GDF15* 3′ UTR variant, rs1054564, results in allele-specific translational repression of GDF15 by hsa-miR-1233-3p

**DOI:** 10.1371/journal.pone.0183187

**Published:** 2017-08-14

**Authors:** Ming-Sheng Teng, Lung-An Hsu, Shu-Hui Juan, Wen-Chi Lin, Ming-Cheng Lee, Cheng-Wen Su, Semon Wu, Yu-Lin Ko

**Affiliations:** 1 Department of Research, Taipei Tzu Chi Hospital, Buddhist Tzu Chi Medical Foundation, New Taipei City, Taiwan; 2 The First Cardiovascular Division, Department of Internal Medicine, Chang Gung Memorial Hospital and Chang Gung University College of Medicine, Taoyuan, Taiwan; 3 Graduate Institute of Medical Sciences, Department of Physiology, College of Medicine, Taipei Medical University, Taipei, Taiwan; 4 Department of Life Science, Chinese Culture University, Taipei, Taiwan; 5 Cardiovascular Center and Division of Cardiology, Department of Internal Medicine and Taipei Tzu Chi Hospital, Buddhist Tzu Chi Medical Foundation, New Taipei City, Taiwan; 6 School of Medicine, Tzu Chi University, Hualien, Taiwan; Universitat des Saarlandes, GERMANY

## Abstract

Growth differentiation factor 15 (GDF15) is a strong predictor of cardiovascular events and mortality in individuals with or without cardiovascular diseases. Single nucleotide polymorphisms (SNPs) in microRNA (miRNA) target sites, also known as miRSNPs, are known to enhance or weaken miRNA-mRNA interactions and have been linked to diseases such as cardiovascular disease and cancer. In this study, we aimed to elucidate the functional significance of the miRSNP rs1054564 in regulating GDF15 levels. Two rs1054564-containing binding sites for hsa-miR-873-5p and hsa-miR-1233-3p were identified in the 3′ untranslated region (UTR) of the GDF15 transcript using bioinformatics tools. Their activities were further characterized by *in vitro* reporter assays. Bioinformatics prediction suggested that miRNA binding sites harboring the rs1054564-G allele had lower free energies than those with the C allele and therefore were better targets with higher affinities for both hsa-miR-873-5p and hsa-miR-1233-3p. Reporter assays showed that luciferase activity was significantly decreased by rs1054564-G-containing 3′ UTRs for both miRNAs (P < 0.05) and was restored by miRNA inhibitors. Comparing the fold suppression of the two miRNAs, only that of hsa-miR-1233-3p showed significant changes between the rs1054564-G- and C-containing 3′ UTRs (P = 0.034). In addition, western blots showed that transfection of both miRNA mimics significantly decreased endogenous GDF15 expression in a melanoma cell line (P < 0.05). Taken together, our findings demonstrate that GDF15 is a target of hsa-miR-873-5p and hsa-miR-1233-3p and that the rs1054564-C allele partially abolishes hsa-miR-1233-3p-mediated translational suppression of GDF15. These results suggest that rs1054564 confers allele-specific translational repression of GDF15 via hsa-miR-1233-3p. Our work thus provides biological insight into the previously reported clinical association between rs1054564 and plasma GDF15 levels.

## Introduction

Growth differentiation factor 15 (GDF15) is a member of the transforming growth factor-β cytokine superfamily and its expression is low in all organs under normal conditions but increases in response to stress signals in adults [1]. GDF15 is secreted by cells in response to ischemia, proinflammatory cytokine stimulation, and oxidative or mechanical stress [1], and it diffuses rapidly via circulation [2]. Circulating GDF15 levels have been used to predict disease progression in cancer, cardiovascular disease, chronic renal and heart failure, and pulmonary embolism [3]. GDF15 is also a strong predictor of cardiovascular, non-cardiovascular, and all-cause mortality in community-dwelling and disease populations [4]. Although GDF15 appears to have anti-inflammatory and antiapoptotic effects in the heart [5], the co-localization of GDF15 with apoptotic markers in active macrophages suggests it may have proinflammatory effects. Thus, it remains unknown whether GDF15 is a simple biomarker or whether it is an active protective or detrimental mediator of cardiovascular events. Previous studies have shown associations between GDF15 levels and genetic polymorphisms, clinical parameters, and levels of circulating metabolic and inflammatory markers, albeit with controversial results [6–11].

MicroRNAs (miRNAs) are a class of single-stranded, endogenous, non-coding RNAs of approximately 22 nt that play vital regulatory roles in animals and plants by targeting mRNAs for degradation or translational repression [12]. It is estimated that an average miRNA has approximately 100–200 target sites, and a large fraction (~30%) of protein-coding genes appear to be regulated by miRNAs. Recent studies have shown crucial correlations between single nucleotide polymorphisms (SNPs) in miRNA-related pathways and many pathological conditions [13–16]. SNPs in microRNA (miRNA) target sites, also known as miRSNPs, in the 3′ untranslated regions (UTRs) of target genes, in particular, represent a specific mode of control of genetic information amplification, whose dysregulation may lead to substantial differences in posttranscriptional gene expression. By definition, miRSNPs in the seed sequence (i.e., the region of base-pairing between nucleotides 2–8 of the miRNA and the complementary sequence in the target mRNA) can create, destroy, or modify miRNA–mRNA binding [17–19], and as a result, these function as gain- or loss-of-function mutations. Whereas gain-of-function mutations in 3′ UTRs create new miRNA target sites and attenuate protein translation, loss-of-function mutations in 3′ UTRs reduce or abolish miRNA–mRNA interactions and augment protein expression. For example, a mismatch in the seed sequence pairing of miR-22 and its target site in the *TNFAIP8* 3′ UTR has been shown to abolish translational repression of *TNFAIP8* [20].

Owing to their potential to alter protein translational efficiency, miRSNPs are likely to contribution to phenotypic variation and disease susceptibility. Several studies have used computational approaches to predict miRSNPs in the genome, and significant associations between these miRSNPs and respective protein levels or related disease traits have been found [20–24]. However, it is difficult to prove that these associations are not instead due to linkage disequilibrium with other SNPs or some other mechanisms. Although increasingly sophisticated computational tools to predict miRSNPs are becoming available, target prediction still remains a major challenge and requires *in vitro* experiments for functional validation.

Among the SNPs near the 3′ UTR of the *GDF15* locus, rs1054564 showed the most significant association with circulating GDF15 levels [25, 26]. The Framingham study also revealed that rs1054564 was associated with cis-gene expression of *PGPEP1* and *LRRC25* in blood cell lines and lower circulating HDL cholesterol levels [9]. Ek et al. [27] further indicated that genetically increased GDF15 levels, such as via *GDF15* SNP rs1054564, directly influence methylation levels at several CpG sites. The aim of this study was to determine if specific miRNAs are capable of regulating GDF15 expression via translational repression.

## Materials and methods

### In silico analyses

MirSNP (http://cmbi.bjmu.edu.cn/mirsnp), a publicly available online database, is a collection of human SNPs in predicted miRNA–mRNA binding sites. Analyses of the miRNA binding sites in the *GDF15* 3′ UTR were performed using microRNA.org (http://www.microrna.org/microrna/home.do) and TargetScan (http://www.targetscan.org/). We used the NCBI database of SNPs (dbSNP; http://www.ncbi.nlm.nih/gov/SNP) to obtain information about genetic variations. Using these bioinformatics tools, we identified two miRNAs, hsa-miR-873-5p and hsa-miR-1233-3p, that potentially bind to a stretch of sequence harboring rs1054564 in the 3′ UTR of *GDF15*. The miRNA–target binding structures and energies were predicted using RNAhybird (http://bibiserv.techfak.uni-bielefeld.de/rnahybird).

### Construct

A genomic DNA fragment of the *GDF15* 3′ UTR was amplified by PCR from one individual who was heterozygous for rs1054564 in our previous association study [26]. The upstream and downstream primers used were 5′-ACTAGCTGCATATGAGCAGTCCTGGTCC-3′ and 5′-AAGCTTCACCACAGGGAACAGTTCAG-3′, which were tagged with the *Spe*I and *Hind*III restriction enzyme sites (underlined), respectively. PCR products were subcloned into the pCR^®^2.1 vector (Invitrogen, Carlsbad, CA, USA) following the manufacturer’s protocol. Plasmid DNA was subsequently isolated from recombinant colonies and sequenced to ensure accuracy. The *GDF15* 3′ UTR inserts were then extracted by *Spe*I/*Hind*III digestion, gel-purified, and subcloned into the *Spe*I/*Hind*III site of pMIR-REPORT^™^ (Ambion) downstream of the firefly luciferase reporter gene. The validity and orientation of the inserts relative to the luciferase gene were confirmed by sequencing.

### Cell culture

Human embryonic kidney HEK293T (from Dr. Tzung-Chieh Tsai) and melanoma A2058 cell lines (Food Industry Research and Development Institute, Taiwan) were maintained in Dulbecco’s modified Eagle’s medium (Invitrogen) supplemented with 10% fetal bovine serum (HyClone Laboratories, Logan, UT, USA), 80 units/ml penicillin, 80 μg/ml streptomycin, and 0.0175 mg/ml l-proline (Sigma).

### Transfection and luciferase assay

Mimics and inhibitors for hsa-miR-873-5p and hsa-miR-1233-3p as well as miRNA negative control #1 were obtained from Ambion (Carlsbad, CA, USA). Cells were plated in a 24-well plate and grown to 80–90% confluence. Firefly luciferase constructs (500 ng) were cotransfected with 20 nM mimic miRNAs with or without Ambion^®^ Anti-miR^™^ miRNA inhibitors into HEK293T cells using Lipofectamine 2000 (Invitrogen). To monitor transfection efficiency, cells were additionally cotransfected with 50 ng pRL-TK (Promega, Madison, WI, USA), which encoded the *Renilla* luciferase. Luminescence was measured 48 h after transfection using a dual-luciferase reporter assay system (Promega). All transfections were performed in triplicate, and data were analyzed by normalizing firefly luciferase activity with that of the *Renilla* luciferase for each sample. Each construct was tested in three independent transfections.

### Western blot

Equal amounts of total cell lysate proteins were loaded, separated by 10% SDS-PAGE, and transferred to polyvinylidene difluoride membranes. Membranes were incubated with primary antibodies against GDF15 (Santa Cruz Biotechnology, Santa Cruz, CA, USA) for 1 h and subsequently with horseradish peroxidase-conjugated secondary antibodies for 1 h. Band densities were detected by ECL chemiluminescence (Amersham Biosciences, Buchs, Switzerland) as described by the manufacturer. Tubulin (Cell Signaling Technology, Beverly, MA, USA) was used as an internal control. Images were scanned with a master imager (Microtek ScanMaker 9800XL, Shanghai, China) and semi-quantified with Photoshop 7.0 (Adobe, San Jose, CA, USA).

### Statistical analyses

All statistical analyses were performed using the SPSS 12.0 statistical software package (SPSS, Chicago, IL, USA). Relative luciferase activity data are presented as means ± SD and were analyzed with Student’s *t*-test. All P-values reported are two-sided. P < 0.05 was considered statistically significant.

## Results

### Rs1054564 is located in the predicted binding sites of hsa-miR-873-5p and hsa-miR-1233-3p in the *GDF15* 3′ UTR

**Fig 1:**
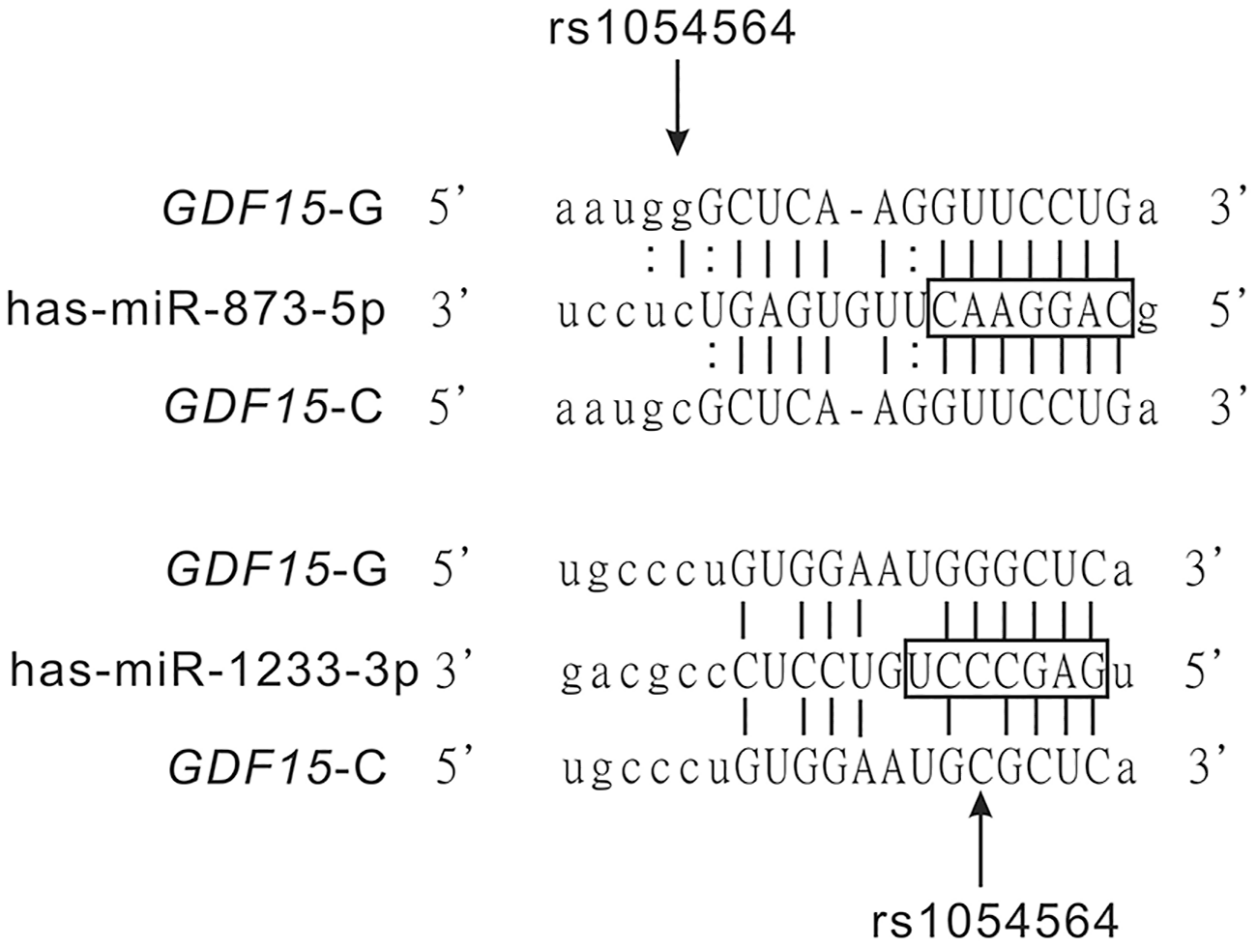
Schematic of *GDF15* mRNA harboring putative miR-873-5p/miR-1233-3p binding sites and SNP rs1054564 G>C in the 3′ UTR. Alignment shows the *GDF15* 3′ UTR variant rs1054564 G>C region with the miRNA hsa-miR-873-5p and hsa-miR-1233-3p motifs. The location of the rs1054564 G>C polymorphism is designated with an arrow in the sequence alignment. Allele G forms a Watson-Crick base-pair with C in the miRNAs (solid line), whereas allele C does not (no line). The seed regions of both miRNAs are designated with an open box. The dots between the base pairs G:U represent GU wobble pairs.

Using a publicly available online database, MirSNP (http://cmbi.bjmu.edu.cn/mirsnp), hsa-miR-873-5p was identified as the only conserved miRNA predicted to bind to the *GDF15* 3′ UTR in the region containing rs1054564 with a good mirSVR score (-0.1265) (Table 1). The prediction indicated an enhanced binding activity based on the rs1054564-G allele adjacent to the 3′ end of the conserved non-seed region of hsa-miR-873-5p (Fig 1). In order to search for other possible miRNAs that could involve rs1054564, we further used two miRNA target prediction tools, MiRanda and TargetScan. A non-conserved miRNA, hsa-miR-1233-3p, was predicted to bind to the *GDF15* 3′ UTR in the region that contains rs1054564 with a low mirSVR score (-0.0096) (Table 1), and rs1054564 was located inside the hsa-miR-1233-3p seed sequence (Fig 1).

**Table 1:** Prediction results of miRNAs targeting the *GDF15* 3′ UTR SNP rs1054564.

Gene	SNP	Allele	miRNA	Conserved^a^	mirSVR^b^	Energy^c^	Score^d^	Seed region
GDF15	rs1054564	**G**	hsa-miR-873-5p	yes	-0.1265	-28.4	97	no
		**C**				-26.0		
		**G**	hsa-miR-1233-3p	no	-0.0096	-27.1	89	yes
		**C**				-21.4		

^a^Conservation information among vertebrates from microRNA.org

^b^mirSVR score of binding site from microRNA.org

^c^Free energy of miRNA–mRNA duplex from RNAhybird

^d^Predicted score of miRNA–mRNA binding by TargetScan. The higher the score the more stable the binding.

### Predicted structures and binding energies of miRNA–target duplexes

To determine the potential of rs1054564 alleles to alter predicted miRNA–mRNA interactions, we conducted *in silico* hybridization between the predicted miRNAs and *GDF15* 3′ UTRs containing the major or minor allele of rs1054564. The affinity between each miRNA and its target sequence can be assessed by computing the minimal free energy of the double-stranded RNA hybrid [28], and this allowed us to compare the stabilities of the miRNA-target interactions among hybrids with different rs1054564 alleles. The program RNAhybrid (http://bibiserv.techfak.uni-bielefeld.de/rnahybrid/) was used to evaluate the Gibbs free energy (ΔG, expressed as kcal/mole) for each rs1054564-containing 21-mer target sequence hybridized to the miRNA of interest. RNAhybrid determined the most favorable hybridization site for each miRNA and subsequently computed the hybridization energy and a potential base-pairing pattern.

**Fig 2:**
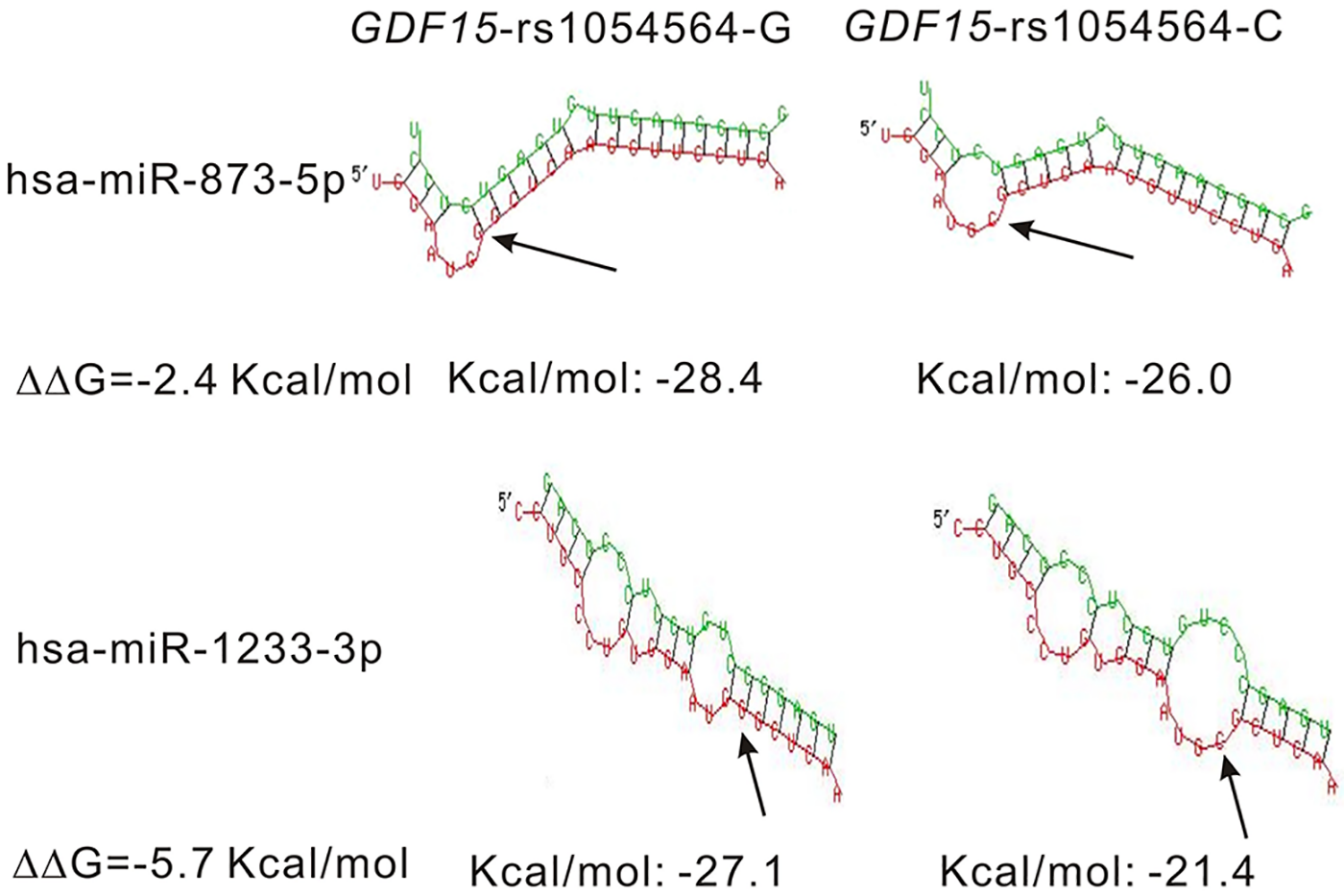
In silico analysis of the pairing of miR-873-5p and miR-1233-3p to the binding site in the 3′ UTR of *GDF15* showing the effect of the minor allele of rs1054564 on the hybrid structures formed between hsa-miR-873-5p/hsa-miR-1233-3p and the *GDF15* 3′ UTR. Allele C disrupts the stem part of the typical stem-loop RNA folding structure. The arrow indicates the SNP site in the *GDF15* 3′ UTR in each folding structure.

The predicted minimal folding energies of the hsa-miR-873-5p- and hsa-miR-1233-3p-target duplexes differed for the rs1054564-G and -C alleles (−28.4 *vs*. −26.0 kcal/mole for the hsa-miR-873-5p-rs1054564-G and -rs1054564-C duplexes respectively, and −27.1 *vs*. −21.4 kcal/mole for the hsa-miR-1233-3p-rs1054564-G and -rs1054564-C duplexes, respectively) (Fig 2). The lower minimal folding energies of the rs1054564-G-containing duplexes indicated more stable binding of hsa-miR-873-5p and hsa-miR-1233-3p to the target mRNA and hence more efficient translational repression of the rs1054564-G-containing *GDF15* transcript. The difference in Gibbs free energy (ΔΔG) between rs1054564-G- and -C-containing hsa-miR-1233-3p–mRNA hybrids (−5.7 kcal/mole) was lower than that between rs1054564-G- and -C-containing hsa-miR-873-5p–mRNA hybrids (−2.4 kcal/mole), suggesting a more pronounced effect of rs1054564 variation on the mRNA-hsa-miR-1233-3p interaction than on the mRNA-hsa-miR-873-5p interaction.

### Functional analyses of rs1054564

**Fig 3:**
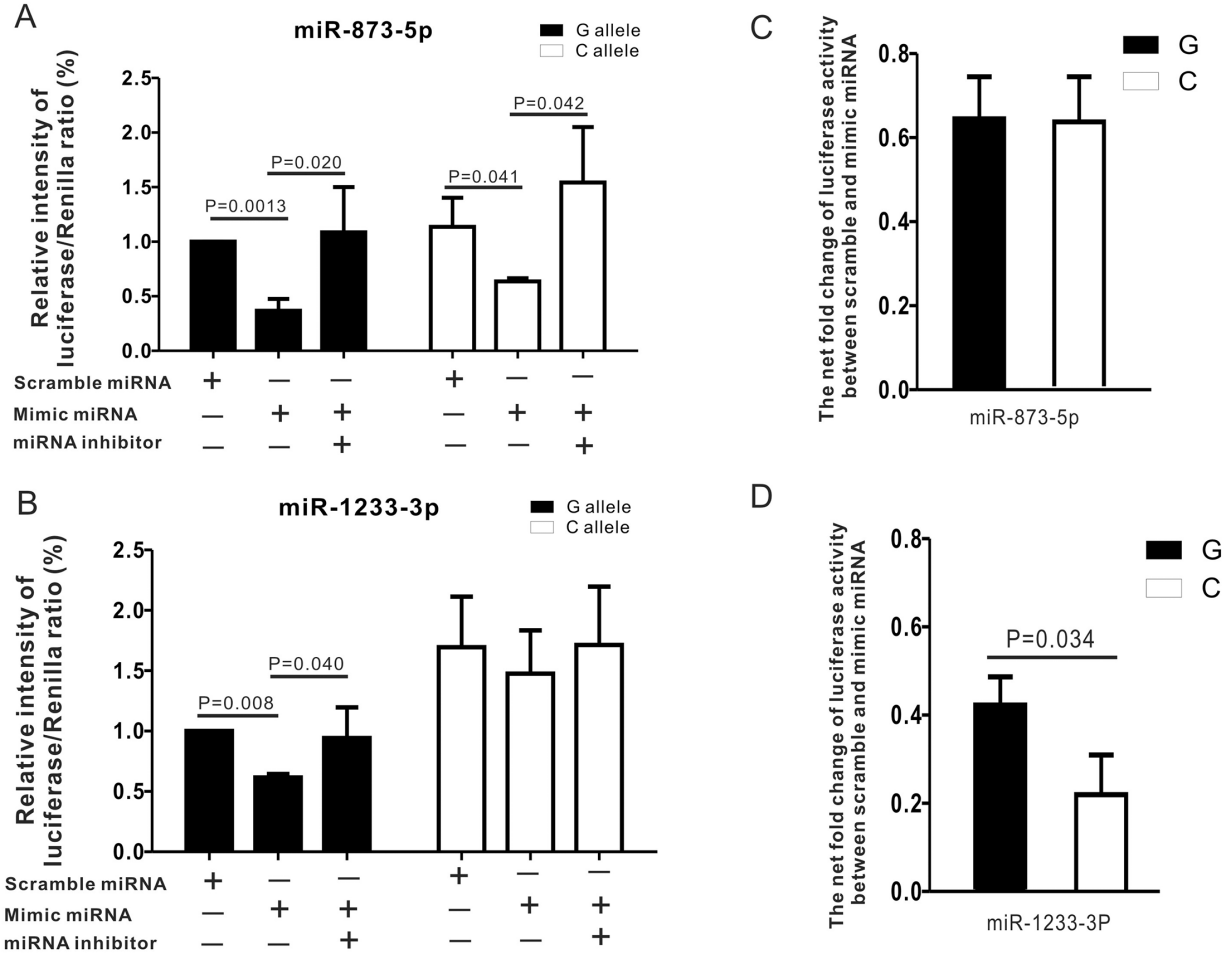
Mimic miRNAs regulate human *GDF15* 3′ UTR expression in human HEK293T cells. Results of luciferase reporter activity were analyzed by Student’s *t*-test and are expressed as mean ± SEMs. (A) Effect of mimic miR-873-5p. (B) Effect of mimic miR-1233-3p. (C) Difference in net suppressive effect of mimic miR-873-5p between rs1054564 alleles G and C. (D) Difference in net suppressive effect of mimic miR-1233-3p between rs1054564 alleles G and C.

To examine whether the rs1054564 variants affected the translational regulation of the GDF15 protein, we generated luciferase reporter constructs containing the *GDF15* 3′ UTR with different rs1054564 alleles. These constructs were designated as pMIR-G and pMIR-C, containing the rs1054564-G and -C alleles, respectively. These constructs were cotransfected with either a scrambled or a mimic miRNA of interest into HEK293T cells. Compared with the results of the scrambled miRNA control, luciferase expression was significantly reduced following transfection with either pMIR-G or pMIR-C in the presence of hsa-miR-873-5p (63.2% decrease for pMIR-C, *P* = 0.041; 64.5% decrease for pMIR-G, *P* = 0.0013) (Fig 3A). However, in the presence of hsa-miR-1233-3p, expression was only reduced following transfection with pMIR-G (42.1% decrease, *P* = 0.008) (Fig 3B). In other words, while hsa-miR-873-5p suppressed luciferase expression of both constructs, resulting in a 1.7% difference in the fold-changes between the constructs (*P* > 0.05) (Fig 3C), hsa-miR-1233-3p only significantly suppressed luciferase expression from pMIR-G, resulting in a 20.1% difference in fold-changes between the two constructs (*P* = 0.034) (Fig 3D). We further used miRNA inhibitors to assess whether the translational suppression was indeed caused by the respective miRNAs, and both miRNA inhibitors fully reversed this suppression. Our data thus indicate that hsa-miR-1233-3p may preferentially target the *GDF15* 3′ UTR carrying the major rs1054564-G allele for translational suppression.

### GDF15 protein levels are regulated by both hsa-miR-873-5p and hsa-miR-1233-3p

**Fig 4:**
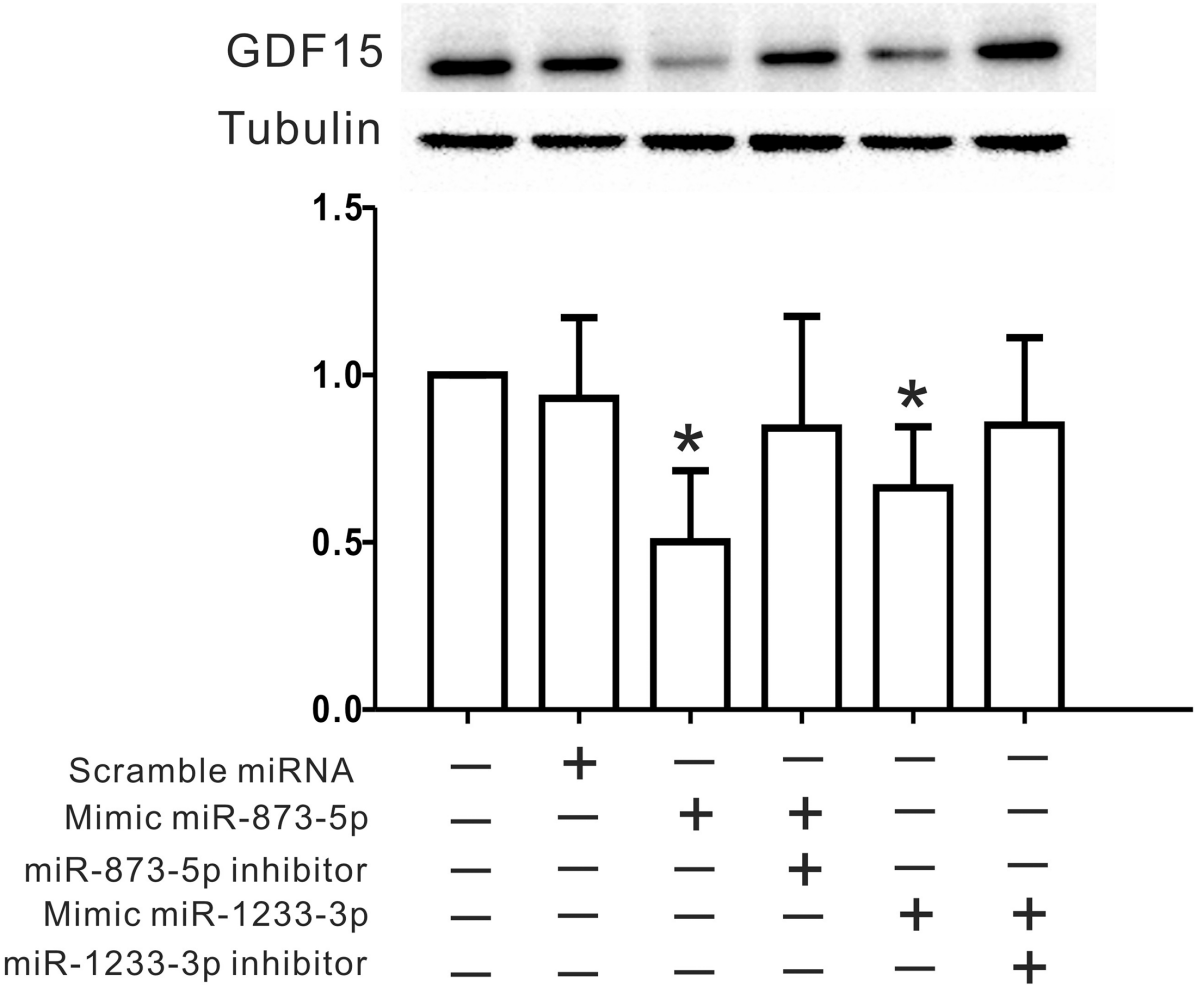
Effect of hsa-miR-873-5p and hsa-miR-1233-3p mimic miRNAs on GDF15 protein expression in human melanoma A2058 cells. Endogenous GDF15 was suppressed by both mimic miRNAs and restored by miRNA inhibitors. Data represent expression levels relative to those of control samples and indicate the mean of three independent experiments; error bars indicate SE. *p*-values were determined by a Student’s *t*-test, and * denotes a *p*-value < 0.05.

To determine the effect of hsa-miR-873-5p and hsa-miR-1233-3p on endogenous GDF15 protein expression, we transfected the two mimic miRNAs into the A2058 melanoma cell line. Western blot showed that both hsa-miR-873-5p and hsa-miR-1233-3p significantly downregulated GDF15 protein levels (42.7% decrease by hsa-miR-873-5p, *P* = 0.018; 26.7% decrease by hsa-miR-1233-3p, *P* = 0.030). This effect was significantly reversed by treatment with corresponding miRNA inhibitors (Fig 4). However, downregulation of GDF15 expression by hsa-miR-873-5p was more effective than that by hsa-miR-1233-3p.

Our findings demonstrate that GDF15 is a target of hsa-miR-873-5p and hsa-miR-1233-3p and that the rs1054564-C allele partially abolishes hsa-miR-1233-3p-mediated translational suppression of GDF15.

## Discussion

In this study, we discovered that both hsa-miR-873-5p and hsa-miR-1233-3p repressed endogenous GDF15 translation in a melanoma cell line, indicating that the *GDF15* transcript is indeed a target of these miRNAs. Moreover, we are the first to report that variants of the SNP rs1054564 differentially regulate hsa-miR-1233-3p-mediated translational repression. Higher luciferase expression and weaker binding to hsa-miR-1233-3p tentatively suggest that individuals carrying the minor rs1054564-C allele may exhibit elevated GDF15 protein expression. Under pathological conditions like chronic systemic inflammation where GDF15 levels are already high [29–31], the presence of this C-allele may predispose carriers to an even greater risk of undesirable disease outcomes and poor prognoses.

Polymorphisms in miRNA target sites can affect the binding efficacy of miRNA. As a result, they can alter target gene expression and may even contribute to the development of certain diseases. In fact, a number of studies have already reported associations between miRSNPs and human diseases [32–38]. Most of these studies focused on miRSNPs within the target sequence complementary to the seed region of the conserved miRNAs, as a mismatch between the two sequences in this region can alter miRNA-target interactions. For instance, Yuan et al. identified an *MMP-9* SNP, rs1056628, that was located in the *MMP-9* 3′ UTR region complementary to the miR-491 seed sequence and that contributed to an increased risk of atherosclerotic cerebral infarction by increasing MMP-9 expression through destruction of a miR-491 target site [34]. Moreover, Saba et al. showed that SNP rs9291296 is located in the 3' UTR of gamma-aminobutyric acid receptor subunit alpha-4 in a region complementary to the seed sequence of miR-26a-5p. This SNP strengthens miR-26a-5p binding by creating a target site and was found to be associated specifically with degenerating neurons, such as prion disease and other neurodegenerative disorders [35].

In the current study, we identified a non-conserved miRNA, hsa-miR-1233-3p, the binding affinity of which was significantly affected by miRSNP rs1054564. Functional data showed that luciferase expression was only significantly reduced by the rs1054564-G allele, implying that the rs1054564-C allele destroys a target site in the *GDF15* 3′ UTR complementary to the seed region of hsa-miR-1233-3p. It is worth noting that hsa-miR-1233-3p may play a significant role in the regulation of GDF15, even though its lower ranking score from the prediction tools reflected a lack of evolutionary conservation. Indeed, two recent studies have identified and validated significant roles of non-conserved miRNAs [37, 39]. Cui et al. [39] showed that the C allele of SNP rs2266788 destroys the miRNA hsa-miR-3201 binding site at in the *APOA5* 3′ UTR, thereby increasing translation of *APOA5* and subsequently increasing plasma APOA5 levels. In addition, Ryan et al. [37] identified a SNP that disrupted a novel binding site for miR-516a-3p, leading to moderate increases in *CXCR2* mRNA and protein expression and increased MAPK signaling that was associated with lung cancer risk. These results indicate that non-conserved miRNAs and genetic polymorphisms can play potentially significant roles in protein regulation.

As found in this study, several previous studies have identified two miRNAs regulating gene expression via overlapping target sites that contain the same SNP [28, 39, 40]. Consistent with our study, Minguzzi et al. found that *MTHFD1L* SNP rs7646 creates a target site complementary to the seed region of miR-197 that contributes to hybrid stability; in contrast, SNP rs7646 is located in a region complementary to the non-seed region of miR-9. Their results demonstrated that rs7646 significantly affects miR-197 binding affinity, causing greater suppression when miR-197 is bound to *MTHFD1L* mRNA containing the G allele rather than the A allele. However, rs7646 did not cause any significant changes in miR-9 binding affinity.

Using computational modeling, we predicted and computed the minimal free energies of the secondary structures of has-miR-873-5p and has-miR-1233-3p when bound to the *GDF15* 3′ UTR containing either a G or C allele at SNP rs1054564. Because a SNP in this sequence could influence the interactions of several miRNAs, the sum of the ΔΔG value was used to assess the impact of this SNP. Landi et al. [41] proposed that the sum of the ΔΔG reflects the influence of a SNP in the interactions between miRNAs and the target sequence. Namely, the larger the sum of the ΔΔG of a SNP, the more likely it is to be a functional SNP. Our results showed that rs1054564-C might significantly change the has-miR-1233-3p binding affinity, causing a loss of the suppressive effect, whereas it results in only a mild change in the has-miR-873-5p binding affinity. Thus, our data provide evidence that *GDF15* is a direct miR-1233-3p target and that the *GDF15*-associated SNP rs1054564 affects miR-1233-3p binding efficacy.

MiR-1233 has been found to play a role in a plethora of diseases. For example, it is a potential biomarker for cancer and cardiovascular disease [42–45], and its overexpression in placenta significantly decreases the proliferation and invasive ability of trophoblasts in patients with hypertensive disorder complicating pregnancy [46]. Interestingly, while its expression changes in opposite directions in patients with gastric cancer and renal cell carcinoma [42, 43], it is consistently upregulated in patients with heart failure and acute pulmonary embolism [44, 45]. As higher GDF15 levels are also found in these patients [47], these results thus imply the existence of a feedback mechanism in which *miR-1233* transcription is increased in order to compensate for elevated GDF15 levels under disease conditions.

Our results clearly indicate that hsa-miR-873-5p represses GDF15 translation regardless of rs1054564 genotype. Although hsa-miR-873-5p and GDF15 levels appeared to be negatively correlated in our *in vitro* assays, elevated hsa-miR-873-5p or GDF15 levels have been previously associated with different stages of cancer-like cell proliferation [48–50], metastasis [49, 51, 52], and chemoresistance [47, 53–55]. One reason for these findings is that hsa-miR-873-5p represses the translation of several proteins in addition to GDF15, and this may in turn increase the transcription or prolong the mRNA stability of *GDF15*. Furthermore, it is possible that hsa-miR-873-5p and GDF15 are often not co-expressed in the same type of cancer. Simply overexpressing miR-873-5p or GDF15 may therefore generate completely different results depending on the cell type-specific machinery and underlying gene expression profiles of the cancer cells in question [49,56]. Nevertheless, our combined results of hsa-miR-873-5p-mediated repression of luciferase and endogenous GDF15 expression in two different cell types suggest that miR-873-5p may represent a novel therapeutic target for the treatment of cancers that exhibit increased activity of the *GDF15* gene.

In addition to cancer, it has been reported that the chromosome 9p21 locus, where *miR-873-5p* resides, is strongly associated with coronary artery disease (CAD) [57–63] and peripheral arterial disease (PAD) [64, 65]. Intriguingly, we have previously showed that GDF15 serves as a prognostic factor for all-cause mortality in diverse human disorders including CAD and PAD [26]. Based on the findings of this study, it is tantalizing to speculate that miR-873-5p participates in CAD and PAD through cis-regulation of GDF15 expression. However, other open reading frames in the vicinity of 9p21, including cyclin-dependent kinase inhibitor 2A/B, may also contribute to these diseases [66]. Future studies are needed to determine whether miR-873-5p plays a role in the cardiovascular system and whether it loses its ability to repress GDF15 expression under pathological conditions.

There are several limitations to our study. First, we could not find a cell line heterozygous for rs1054564 to examine the allelic differences in GDF15 translation. Second, we could not detect endogenous hsa-miR-873-5p and hsa-miR-1233-3p expression in the cell lines used in this study and therefore could not address their modes of interaction in greater detail. Third, a suitable animal model was not available, hampering further investigation. Finally, the mechanism by which hsa-miR-873-5p and hsa-miR-1233-3p regulate GDF15 translation is not yet fully understood and requires further elucidation.

## Conclusions

Our work delineates a comprehensive framework for miRNA-mediated translational repression of GDF15. We propose that in a certain percentage of the human population, the rs1054564-C mutation weakens the hsa-miR-1233-3p binding site in the *GDF15* 3′ UTR and thereby increases the chances for higher GDF15 protein expression. This upregulation might at least partially account for the strong associations between rs1054564 and elevated GDF15 levels reported in previous study [26]. Further investigations regarding the interactions between the studied miRNAs and GDF15 levels and their involvement in the progression of cardiovascular diseases and cancers should be interesting and may help to uncover novel therapies against related complications.
